# Spatial-temporal comparison of Eph/Ephrin gene expression in ocular lenses from aging and knockout mice

**DOI:** 10.3389/fopht.2024.1410860

**Published:** 2024-06-04

**Authors:** Peter N. Huynh, Catherine Cheng

**Affiliations:** School of Optometry and Vision Science Program, Indiana University, Bloomington, IN, United States

**Keywords:** EphA2, Ephrin-A5, RT-qPCR, epithelium, lens fibers

## Abstract

Cataracts, defined as any opacity in the transparent ocular lens, remain the leading cause of blindness and visual impairment in the world; however, the etiology of this pathology is not fully understood. Studies in mice and humans have found that the EphA2 receptor and the ephrin-A5 ligand play important roles in maintaining lens homeostasis and transparency. However, due to the diversity of the family of Eph receptors and ephrin ligands and their promiscuous binding, identifying functional interacting partners remains a challenge. Previously, 12 of the 14 Ephs and 8 of 8 ephrins in mice were characterized to be expressed in the mouse lens. To further narrow down possible genes of interest in life-long lens homeostasis, we collected and separated the lens epithelium from the fiber cell mass and isolated RNA from each compartment in samples from young adult and middle-aged mice that were either wild-type, *EphA2^–/–^* (knockout), or ephrin-A5*^–/–^*. Reverse transcription quantitative polymerase chain reaction (RT-qPCR) was implemented to compare transcript levels of 33 Eph and ephrin gene variants in each tissue compartment. Our results show that, of the Eph and ephrin variants screened, 5 of 33 showed age-related changes, and 2 of 33 showed genotype-related changes in lens epithelium. In the isolated fibers, more dynamic gene expression changes were observed, in which 12 of 33 variants showed age-related changes, and 6 of 33 showed genotype-related changes. These data allow for a more informed decision in determining mechanistic leads in Eph-ephrin-mediated signaling in the lens.

## 1 Introduction

The ocular lens is a transparent and avascular structure in the anterior chamber of the eye that facilitates the fine focusing of light onto the retina. Cataracts, describing any opacification of the lens, remain the leading cause of blindness in the world (1). While cataract surgery is a common medical treatment, access to adequately equipped medical professionals and facilities remains difficult for many people (1). To better treat and prevent these afflictions, a deeper understanding of how the lens maintains its homeostasis, transparency, and accommodative ability is required.

Recent studies have highlighted the importance of the Eph-ephrin signaling pathway in lens transparency (2–7). Erythropoietin-producing hepatocellular (Eph) receptors make up the largest family of receptor tyrosine kinases and facilitate diverse signaling with their endogenous ligands, the ephrins. Ephs are divided into two families, EphAs and EphBs, and likewise ephrins are divided into ephrin-As and ephrin-Bs. Generally, ephrin-As bind to EphA receptors, and ephrin-Bs bind to EphBs (8, 9). However, cases of ephrin-As binding to EphBs and ephrin-Bs binding to EphAs have been observed as instances of inter-family crosstalk (10, 11). EphA and EphB receptors are encoded by *Epha* and *Ephb* genes, while ephrin-A and ephrin-B ligands are encoded by *Efna* and *Efnb* genes, respectively. Ephs and ephrins facilitate a wide assortment of cellular and developmental processes, including cell morphology, migration, adhesion, and differentiation, resulting in diverse phenotypes when these pathways are dysregulated (12, 13).

Currently, 16 Ephs and 9 ephrins have been identified in total, with 14 Ephs and 8 ephrins present in mice and humans. Two members of these families, the receptor EphA2 and the ligand ephrin-A5, have been targets of interest in lens research due to known mutations linked to both congenital and age-related cataracts in human patients (6, 14–18). The phenotypes manifested from mutations of *EPHA2* and *EFNA5* in human patients are diverse, and the cellular mechanisms involved remain unclear. While EphA2 and ephrin-A5 are known binding partners in other tissues, this receptor and ligand pair are not exclusive binding partners and are spatially segregated in the ocular lens, suggesting their primary binding partners are other ephrins and Ephs (5, 19). In the endeavor to identify relevant binding partners to EphA2 and ephrin-A5, the number of permutations between Ephs and ephrins, and their uncharacterized lens distribution present a challenge in prioritizing targets to investigate.

The lens is composed of two cell types, a monolayer of epithelial cells covering the anterior hemisphere and a bulk mass of fiber cells. Our previous work revealed that loss of EphA2 or ephrin-A5 in mouse lenses lead to either mild nuclear cataracts at the center of the lens or anterior cataracts, respectively. At a cellular level, *Epha2* knockout (*^–/–^*; KO) lenses display misaligned equatorial epithelial cells, a disrupted fulcrum, and disorganized fiber cells (3, 5, 19–22). Additionally, it was observed that *Epha2^–/–^* also results in smaller, more spherical lenses with reduced refractive power and degraded optical quality and decreased proliferation of lens epithelial cells (23, 24). Ephrin-A5 (*Efna5*) knockout lenses develop anterior polar cataracts due to disruption of cell-cell adhesion via E-cadherin and β-catenin mislocalization leading to epithelial-to-mesenchymal transition (EMT) in anterior epithelial cells (3, 5).

While prominent cataract phenotypes and epithelial cell changes are observed in these *Epha2* and *Efna5* knockout mice, the specific downstream signaling and binding partners of these targets have not yet been elucidated in the lens. Due to the promiscuous nature of Eph-ephrin binding, the number of permutations of receptor-ligand combinations presents a formidable barrier to identify binding partners of interest (10, 25, 26). In the endeavor to narrow targets, a concerted effort to identify the Ephs and ephrins that are present in the mouse lens found that 12 of 14 known Ephs and 8 of 8 known ephrins were expressed (27). Here, the next step of this search is presented, in which these previously observed Ephs and ephrins are investigated in a quantitative manner. mRNA transcripts of each target were measured in isolated lens epithelium or fibers to identify targets that significantly change with age or disruption of *Epha2* or *Efna5* as potential targets of interest from a functional and geographical standpoint.

## 2 Materials and methods

### 2.1 Animals

Mice were maintained in accordance with an approved Institutional Animal Care and Use Committee (IACUC) protocols (#21-010 and #24-002) and the Guide for the Care and Use of Laboratory Animals by the National Institutes of Health (NIH). Generation of *Epha2^–/–^* and *Efna5* ^–/–^ mice was previously described (28, 29). All mice were maintained in C57BL/6J backgrounds with wild-type beaded filament structural protein 2 (*Bfsp2*; CP49) genotypes, as *Bfsp2^–/–^* mice exhibit disruptions in the lens fiber cytoskeleton, gap junctions, and ionic homeostasis (3, 30). *Bfsp2* mutations occur spontaneously across various inbred strains, including 129/SvJ, 129/OLa, and FVB/N mice, and have been linked to cataracts in both humans and mice (31–34). Therefore, the wild-type *Bfsp2* gene was validated in these mice prior to investigating cataract mechanisms. Male and female mice ranging from 6-9 weeks (young adult; Y) and 7 months (middle-aged; M) were used for these studies. Littermates were used for comparison between wild-type and KO samples.

### 2.2 RNA isolation

RNA was isolated from epithelium and fiber cell mass fractions using our previous protocol (35). Briefly, samples were collected from at least three different mice of each genotype and each age for RNA isolation. For *Efna5^–/–^* mice, the lenses can present with obvious anterior cataracts. *Efna5^–/–^* mice were excluded if the lenses had obvious cellular defects to prevent detection of gene expression changes that are downstream of EMT in the KO lens epithelial cells (3, 5). Briefly, lenses were carefully dissected from freshly enucleated eyes. To isolate lens epithelium, the collagenous basement membrane around the lens, the capsule, was gently peeled from the lens posterior. The epithelial cells are well-adhered to the lens capsule (3, 35, 36). The remaining fiber mass was not further separated as the organelle-free nuclear fibers are presumed to contain little to no mRNA. Autologous pairs of capsules with attached epithelial cells or fiber cell bulk masses were pooled and homogenized into 400 µL of cold TRIzol (Invitrogen; Waltham, MA, USA; Cat # 15596026). Samples were incubated for 30 minutes at room temperature. For phase separation, 200 µL of chloroform (Alfa Aesar; Ward Hill, MA, USA; Cat # 22920) was added and the samples were shaken vigorously for 15 seconds by hand. Samples were incubated at room temperature for 15 minutes and centrifuged at 14,000*g* for 15 minutes at 4°C. The aqueous phase was transferred to RNase-free tubes (USA Scientific; Ocala, FL; Cat # 1615-5500), and 1 equivalent volume of 200 proof ethanol (Fisher Scientific; Waltham, MA, USA; Cat # BP2818500) was added. Samples were transferred to ‘RNA Clean and Concentrator-5’ kit columns (Zymo Research; Tustin, CA, USA; Cat # ZR1013), and RNA was isolated using manufacturer instructions, with the omission of RNA Binding Buffer. RNA concentrations were measured using a NanoDrop One™ (Thermo Scientific; Waltham, MA, USA; Cat # ND-ONE-W).

### 2.3 Reverse transcription polymerase chain reaction

RNA samples were reverse transcribed to cDNA using a SuperScript™ IV VILO™ polymerase (Invitrogen; Cat # 11756010), according to manufacturer instructions. RNA transcripts were reverse transcribed using the commercial kit primers containing a mix of random and oligo(dT) primers (37). Briefly, 2000 ng of RNA was added to 4 µL of 5X VILO™ reaction mix and brought to a total of 20 µL with molecular grade water (Fisher Scientific; Cat # BP2819-1). The samples were thermocycled for 1 step of annealing at 25°C for 10 minutes, 1 step of reverse transcription at 50°C for 10 minutes, followed by 1 step of enzyme inactivation at 85°C for 5 minutes. These experiments were performed using a MiniAmp Thermal Cycler (Applied Biosystems; Waltham, MA, USA; Cat # A37834). cDNA content was assumed to be a 1:1 conversion from starting RNA. cDNA samples were divided into lots so that no sample would exceed 5 freeze-thaw cycles. cDNA lots were stored at -80°C until use.

### 2.4 Quantitative polymerase chain reaction

For each reaction, 5 ng of cDNA was amplified using standard TaqMan probe conditions. Briefly, forward and reverse primers (900 nM each), a TaqMan probe (250 nM), and TaqMan Fast Advanced Master Mix (ThermoFisher Scientific; Cat # 4444557) were mixed to a final reaction volume of 10 µL. TaqMan primers and probes details are listed in **Supplementary Table S1**. Plates were sealed using MicroAmp™ optical adhesive film (Applied Biosystems; Cat # 4360954). PCR samples were thermocycled for 1 step of uracil-N-glycosylase (UNG) inactivation at 50°C for 2 minutes, 1 step of denaturation at 95°C for 2 minutes, followed by 45 cycles of denaturation at 95°C for 1 second and annealing at 60°C for 20 seconds. Samples were cycled using a QuantStudio 3 Real-Time PCR System (96-well, 0.1 mL format; Applied Biosystems; Cat # A28567). Custom TaqMan probes and primers were designed using Primer Express 3.0.1 (Applied Biosystems; Cat # 4363991), spanning exon-exon junctions where possible. Quantification cycle (C_q_) values were compared to the reference gene, peptidylprolyl isomerase a (*Ppia*). *Ppia* was chosen from a screen of 32 potential reference genes supplied in a TaqMan Array Mouse Endogenous Control Plate (Applied Biosystems; Cat # 4426696).

### 2.5 *Epha7* primer design

*Epha7* has 6 variants, none of which have any regions unique to a given variant within the coding sequence. Therefore, a combinatorial approach was taken to selectively amplify each variant (**Supplementary Figure S1**). Exons 8 and 9 are conserved across all 6 variants, so a single probe was designed to target a region within exon 8 (Variant 1; 1868–1882; NM_010141). A 15-nucleotide sequence at the 5’ end of exon 7 is present in variants 1-3, but not in 4-6 (Variant 1; 1838-1852). Exon-spanning forward primers (referred to as forward A and B) were targeted towards this site to differentiate variants into two sets. Reverse primers were designed at the exon 9-10 junction, as exon 10 had variations that clustered the 6 variants into 3 pairs (reverse primers 1-3). Using the 6 permutations of forward primers A and B with reverse primers 1-3, each of the 6 variants could be specifically targeted (**Supplementary Table S1**). Custom primers were validated on mouse brain samples (data not shown). Further validation was performed only on EphA7 due to every sample yielding signal indistinguishable from noise. Other custom probes were presumed effective based on effective amplification, PrimerExpress 3.0.1 (Applied Biosystems) scoring, and Primer BLAST comparison.

### 2.6 Qualitative polymerase chain reaction and sequencing

For each reaction, 20 ng of cDNA was amplified using Quick-Load Taq 2X Master Mix (New England Biolabs; Ipswich, MA, USA; Cat # M0271) and 200 nM of each primer, following the manufacturer’s protocol (25). Primers and amplicon information are listed in **Supplementary Table S1**. Reactions were performed using the manufacturer-recommended mix composition at a final reaction volume of 25 µL. PCR samples were thermocycled for 1 step of initial denaturation at 95°C for 30 seconds, followed by 45 cycles of denaturation at 95°C for 30 seconds, annealing at 53°C or 54°C for 30 seconds, and elongation at 68°C for 60 seconds per kilobase. A final extension step at 68°C for 5 minutes followed. Samples were separated using a 1% agarose gel supplemented with GelGreen Nucleic Acid Gel Stain (Biotium; Fremont, CA, USA; Cat # 41005). Bands were cut and isolated using the Qiaquick Gel Extraction Kit (Qiagen; Hilden, Germany; Cat # 28704) following manufacturer instructions. The isolated amplified products were sent to Quintara Biosciences (Cambridge, MA, USA) for Sangar sequencing following Quintara sample preparation guidelines.

### 2.7 qPCR data analysis

QuantStudio 3 readings were interpreted using QuantStudio Design & Analysis Software 2.6.0 (Thermo Fisher Scientific). A signal threshold of 0.3 was used to determine quantification cycle (C_q_) values (31, 32). ΔC_q_ values were determined using a custom R script and analyzed using GraphPad Prism version 9.0.0 (121) (GraphPad Software, LLC; Boston, MA, USA). Two-way ANOVA followed by multiple comparison correction by controlling for false discovery rate was implemented. A two-stage linear step-up procedure of Benjamini, Krieger, and Yekutieli was used with a Q value (false discovery rate) of 0.05. A noise threshold of ΔC_q_ = 11.9 was determined using *Epha2* TaqMan probe readings from *Epha2* ^–/–^ mice, taking the lowest (strictest) ΔC_q_ value obtained from this data set. Data sets with more than 2 biological test groups exhibiting a mean above this threshold were considered non-specific amplification and were not included in statistical analysis. *Epha2* and *Efna5* readings in their respective knockouts were tested using multiple unpaired t-tests followed by a two-stage step-up method of Benjamini, Krieger, and Yekutieli with a Q value of 0.05.

## 3 Results

Using traditional, qualitative PCR, 12 of 14 Ephs and 8 of 8 ephrins were previously detected in the lenses of young adult wild-type, *EphA2^–/–^*, and *Efna5^–/–^* mice (27). Unfortunately, the large number of genes expressed did not sufficiently narrow down targets of interest to pursue. Thus, a reverse transcription quantitative polymerase chain reaction (RT-qPCR) method was implemented to determine whether any Eph or ephrin genes are changed by age and/or genotype with considerable magnitude. While qPCR results are commonly presented as 2^–ΔΔCq^, this relies on normalizing readings to a reference group. In this experimental design, there are multiple comparisons that utilize more than a single reference group. Moreover, using ΔC_q_ values allows us to compare relative expression levels between genes rather than normalizing to a control group per gene. Therefore, the majority of data presented are shown as ΔC_q_ values, and it should be noted that lower ΔC_q_ values indicate higher transcript levels.

### 3.1 Establishing an appropriate endogenous control

To appropriately control for well-to-well variation, an internal reference gene was used for each sample. Thirty-two potential reference genes were screened and assessed based on quantification cycle (C_q_) and standard deviation (**Supplementary Figure S2**). Probes and primers for the target genes were pre-aliquoted into a pre-configured 96-well plate containing 16 commonly used reference genes and 16 mouse orthologs of human genes shown to be constitutively expressed in mice (Applied Biosystems; Cat # 4226694). As this screen was in search of endogenous controls, these samples did not include an internal reference gene and were quality controlled based on the passive reference dye, ROX. Therefore, results are shown and analyzed as C_q_ values rather than ΔC_q_.

Of the targets screened, the 18s ribosomal RNA was the highest expressing (lowest C_q_) reference gene with a mean C_q_ (M_Cq_) = 10.4 (SD = 0.79). However, 18s was not chosen as the internal control to avoid depletion of reaction reagents due to its rapid amplification. The next cluster of moderately-expressed targets amplified with C_q_ values between 19.0-22.0. This group consisted of ß-actin (*Actb*), mitochondrially encoded ATP synthase 6 (*ATP6*), glyceraldehyde-3-phosphate dehydrogenase (*Gapdh*), and peptidylprolyl isomerase A (*Ppia*). Previous studies showed that disruption of EphA2 can dysregulate actin distribution and alter cytoskeletal morphology (20). Thus, *Actb* was eliminated as a candidate control despite being a commonly used control. Between *ATP6* (M_Cq_ = 21.5, SD = 1.07), *Gapdh* (M_Cq_ = 20.0, SD = 0.61), and *Ppia* (M_Cq_ = 20.5, SD = 0.39), *Ppia* exhibited the smallest standard deviation. The ribosomal protein L37a (*Rpl37a;* M_Cq_= 25.8, SD = 0.40) also had a comparable standard deviation but crossed the detection threshold at a later amplification cycle. Ultimately, *Ppia* was chosen as the endogenous control due to its earlier amplification and tighter standard deviation. The distribution of *Ppia* across all tested samples are compiled in **Supplementary Figure S3**.

### 3.2 Preliminary qPCR validation with knockout samples

The nature of the *Epha2* and *Efna5* knockout mice results in successfully disrupted protein products; however, portions of the gene remain in the mRNA transcripts. The *Epha2* gene is disrupted with the insertion of a neomycin resistance (*neo*) cassette at a HindIII restriction site located in exon 5 at positions 1410–1415 (NM_010139.3), previously reported at position 1372 (NM_010139), and a downstream XbaI site. This is predicted to produce a non-functional protein truncated after Arg426, between the two extracellular fibronectin domains (28). The *Efna5* knockout uses a similar *neo* insertion technique, targeting a BamHI site at positions 249-254 in exon 1 and an EcoRI restriction site at positions 691-696 in exon 2 (NM_207654.3), replacing amino acids 1–128 (29).

Custom *Epha2* probes were designed to amplify a 65-base-pair (bp) amplicon from positions 1405–1469 (NM_010139.3). The forward primer bound at positions 1405–1422 (**Supplementary Table S1**), spanning the HindIII restriction site. This *Epha2* primer set should not amplify the template if the *neo* cassette is present, and the TaqMan probe was designed to bind 3’ of the restriction site in a presumably deleted portion. However, the custom *Epha2* probe yielded a positive signal in 2 of the 12 *Epha2^–/–^* samples. These readings were used to determine a noise threshold of ΔC_q_ = 11.9, denoting any higher quantification cycles as non-specific signal.

Custom *Efna5* probes and primers were designed to amplify a 70 bp amplicon from positions 831-900 (NM_207654.3). This TaqMan probe spanned exons 4 and 5, binding from positions 860-876. Although this portion of the gene was 3’ of the *neo* insertion, this position was targeted to differentiate *Efna5* variant 2 from variants 1 and 3, which are identical within the coding sequence and only differ in the 5’ untranslated region (UTR). Surprisingly, this probe produced a positive signal from all *Efna5^–/–^* samples, so the knockout was further investigated using Sangar sequencing.

Sequencing results from *Efna5^–/–^* isolated lens fiber samples revealed that exon 2 of the *Efna5* gene was excised, resulting in an out-of-frame deletion from bases 432–724 (amino acids 43–139) (data not shown). This was not expected based on the information available from the original paper describing the construction of the knockout (29). Importantly, exon 2 contains almost the entirety of the receptor binding ectodomain (amino acids 30-159) that is conserved through ephrin-A ligands (Conserved Domain Database: cd10425) (38). While the protein products are non-functional, amplifiable transcripts are still produced and thus, can be detected using RT-qPCR. These knockout readings were not included in our analyses.

### 3.3 Biological groups, target selection, and expression parameters

To investigate gene expression differences related to age and *Epha2* or *Efna5* knockouts, 4 sample groups were used per strain: young-adult wild-type (Y-WT), middle-aged wild-type (M-WT), young-adult knockouts (Y-KO), and middle-aged knockouts (Y-KO). Age-related changes were determined by Y-WT vs. M-WT and Y-KO vs. M-KO comparisons. Genotype-related changes were determined by Y-WT vs. Y-KO and M-WT vs. M-KO. Transcript measures were not compared between tissue compartments nor between strains. Y-WT vs M-KO and M-WT vs Y-KO were not considered useful comparisons due to the confounding effects of both age and genotype. Eph and ephrin targets were chosen based on previous qualitative PCR results (27). Targets and variants that were absent from the lens from the initial screen were not quantitated in this current study.

Expression levels were further divided into three categories of high, moderate, and low expression based on their ΔC_q_ values. While the noise threshold was determined via the *Epha2* knockout, the lowest ΔC_q_ value in the dataset was used to determine a maximum expression value. This lowest ΔC_q_ value also belonged to an *Epha2* reading from *Efna5^–/–^* fibers, at ΔC_q_ = 2.9. Using a ΔC_q_ range of 2.9 – 11.9, the readings were binned into approximate categories using a step size of 3. Using *Epha2* in the fibers (M_ΔCq_ = 3.9) and *Efna5^V1/3^* in the epithelium (M_ΔCq_ = 4.4) as high-expression landmarks, samples were categorized into high expression (M_ΔCq_ < 6.0), moderate expression (6.0 ≤ M_ΔCq_ < 9.0), low expression (9.0 ≤ M_ΔCq_ < 11.9), and noise (M_ΔCq_ ≥ 11.9) categories. These thresholds were based on previous observations that EphA2 is predominantly expressed in the fibers while ephrin-A5 is predominantly expressed in epithelial cells (3).

### 3.4 Eph expression changes in the aging lens epithelium

**Figure 1:**
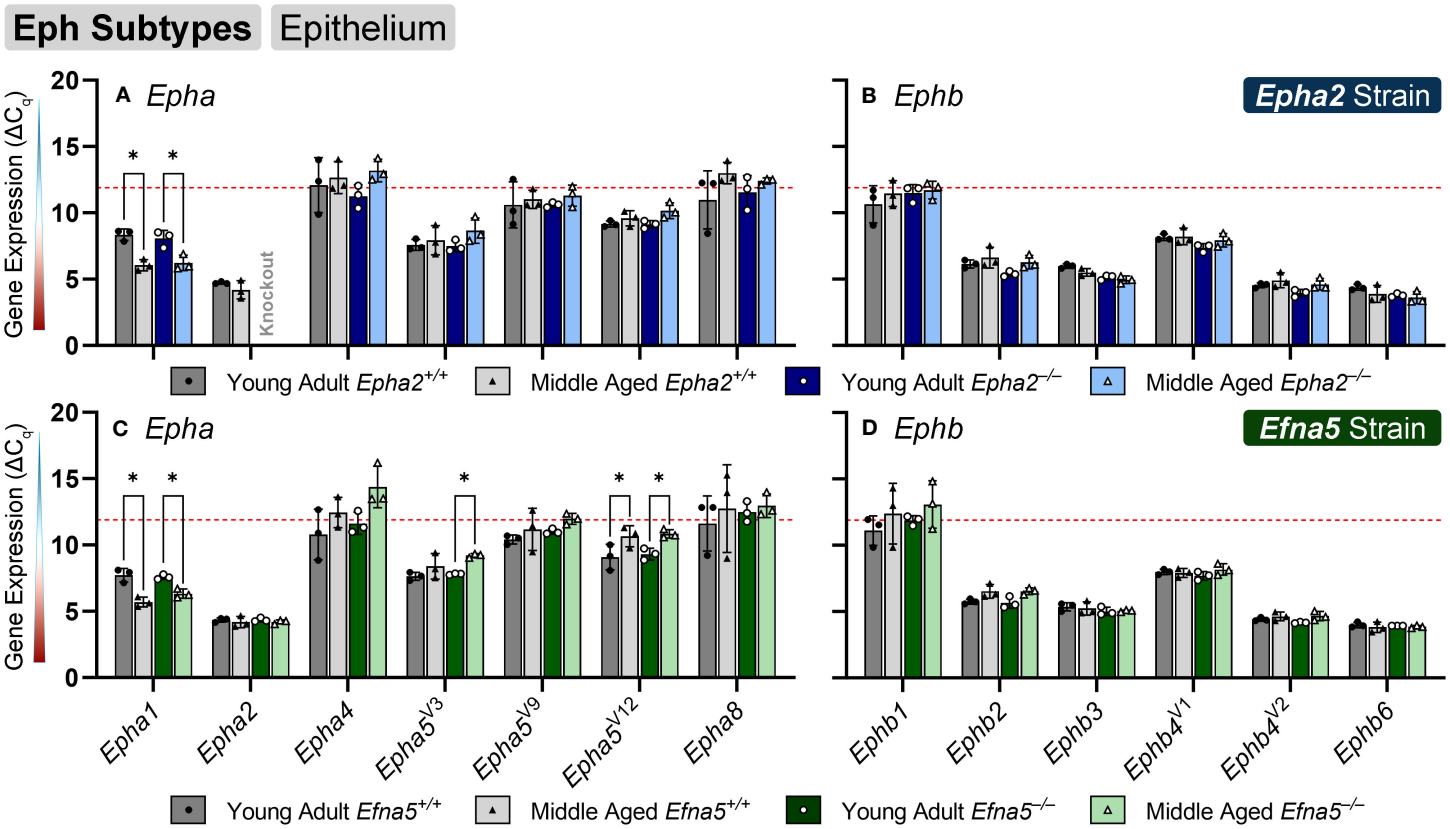
Eph subtypes in lens epithelium. *Epha* and *Ephb* transcripts from lens epithelial cells displayed as ΔC_q_ values. Lower ΔC_q_ values indicate higher expression. *Epha* and *Ephb* gene expression from the *Epha2* strain (panels **A**, **B**; top row; blue palette) and *Efna5* strain (panels **C**, **D**; bottom row; green palette) are displayed separately. Wild-type animals are indicated by filled symbols and grayscale bars, while knockout animals are indicated by hollow symbols and colored bars. Young-adult mice are represented by darker-shaded bars while middle-aged mice are shaded lighter. Significant changes were observed in *Epha1*, *Epha5^V3^*, and *Epha5^V12^* with age. Statistically significant changes with ΔC_q_ values in the noise range (ΔC_q_ ≥ 11.9), as indicated by the red dotted line, were not considered meaningful hits. Two-way ANOVA or multiple unpaired t-tests were used followed by multiple comparison correction. Benjamini-Krieger-Yekutieli-adjusted p-values (q) < 0.05 are considered discoveries and are indicated by asterisks (*).

Within the lens epithelium, *Epha1*, *Epha5^V3^*, and *Epha5^V12^* changed significantly with age regardless of genotype (**Figures 1A, C**). *Epha1* increased with age regardless of genotype in both strains (*Epha2* and *Efna5*; Y-WT vs. M-WT; Y-KO vs. M-KO). A genotype-dependent change was seen in *Epha5^V3^*, where expression levels increase with age only in the *Efna5^–/–^* samples (*Efna5;* Y-KO vs. M-KO). In contrast, a strain difference is observed with *Epha5^V12^* as expression decreased with age in both wild-type and *Efna5^–/–^* lens epithelium (*Efna5;* Y-WT vs M-WT; Y-KO vs. M-KO), but not between the matched samples in the *Epha2* strain. *Epha2* did not show appreciable change in either strain (**Table 1**). *Epha3*, *Epha4*, *Epha5^V9^*, *Epha5^V14^*, *Epha6*, *Epha7^V1-6^*, and *Epha8* were all either in the noise range or did not produce readable signal due to low transcript levels.

**Table 1:** Lens epithelium dynamics summary.

Expression Change Summary	*Epha2* Lens Epithelium				*Efna5* Lens Epithelium			
	Y WT vs M WT	Y KO vs M KO	Y WT vs Y KO	M WT vs M KO	Y WT vs M WT	Y KO vs M KO	Y WT vs Y KO	M WT vs M KO
***Epha1***	▲	▲	** **	** **	▲	▲	** **	** **
***Epha2***	** **	** **	** **	** **	** **	** **	** **	** **
***Epha5^V3^***	** **	** **	** **	** **	** **	▼	** **	** **
***Epha5^V12^***	** **	** **	** **	** **	▼	▼	** **	** **
***Ephb1***	** **	** **	** **	** **	** **	** **	** **	** **
***Ephb2***	** **	** **	** **	** **	** **	** **	** **	** **
***Ephb3***	** **	** **	** **	** **	** **	** **	** **	** **
***Ephb4^V1^***	** **	** **	** **	** **	** **	** **	** **	** **
***Ephb4^V2^***	** **	** **	** **	** **	** **	** **	** **	** **
***Ephb6***	** **	** **	** **	** **	** **	** **	** **	** **
***Efna1***	** **	** **	** **	** **	** **	** **	** **	** **
***Efna2***	** **	▼	** **	** **	▼	▼	** **	** **
***Efna3^IsoA/C^***	** **	** **	▲	** **	** **	** **	** **	** **
***Efna5^V1&3^***	** **	▼	▲	** **	** **	** **	** **	** **
***Efna5^V2^***	** **	** **	** **	** **	** **	** **	** **	** **
***Efnb1***	** **	** **	** **	** **	** **	** **	** **	** **
***Efnb2***	** **	** **	** **	** **	** **	** **	** **	** **

A summary table of significant gene expression hits in epithelial cells across *Epha2* and *Efna5* strains. Young-adult (Y) vs. middle-aged (M) comparisons (columns 1 and 2 in each set) indicate age-related changes, while wild-type (WT) vs knockout (KO) comparisons in columns 3 and 4 indicate a genotype-related change. Gene upregulation (▲) is indicated in black, while downregulation (▼) is shown in red. Genes that did not change across any of the characterized groups are not included in the table.

While no changes were observed in any of the quantitated *Ephb* genes in the lens epithelium (**Figures 1B, D**), *Ephb2*, *Ephb3*, and *Ephb4^V1^* were moderately expressed, while *Ephb4^V2^* and *Ephb6* were highly expressed (**Table 2**). Although both mouse strains are backcrossed to the C57BL/6J background, the two strains are maintained separately and not intermixed. Due to the separate maintenance of these inbred colonies, some genetic drift, and therefore some endogenous strain-related changes are expected. This highlights the necessity of using littermate controls for experiments. Overall, the strains are similar, but strain-dependent differences are evident and discussed below. Of the 14 characterized Ephs in the mouse, only 3 showed notable changes in the epithelium.

**Table 2:** Lens epithelium expression table.

Mean ΔC_q_ Summary	*Epha2* Lens Epithelium								*Efna5* Lens Epithelium							
	Y WT		M WT		Y KO		M KO		Y WT		M WT		Y KO		M KO	
	Mean	St.Dev.	Mean	St.Dev.	Mean	St.Dev.	Mean	St.Dev.	Mean	St.Dev.	Mean	St.Dev.	Mean	St.Dev.	Mean	St.Dev.
***Epha1***	**8.34**	0.428	**6.05**	0.427	**8.06**	0.619	**6.22**	0.585	**7.73**	0.505	**5.69**	0.374	**7.58**	0.182	**6.33**	0.346
***Epha2***	**4.73**	0.087	**4.19**	0.681	In Noise Range		In Noise Range		**4.35**	0.163	**4.19**	0.423	**4.36**	0.151	**4.20**	0.152
***Epha3***	**11.04**	1.764	In Noise Range		**11.39**	0.365	In Noise Range		In Noise Range		In Noise Range		In Noise Range		In Noise Range	
***Epha4***	In Noise Range		In Noise Range		**11.25**	0.851	In Noise Range		**10.79**	1.912	In Noise Range		**11.63**	0.838	In Noise Range	
***Epha5^V3^***	**7.58**	0.413	**7.93**	1.103	**7.48**	0.435	**8.65**	0.949	**7.64**	0.300	**8.39**	0.956	**7.81**	0.057	**9.20**	0.156
***Epha5^V9^***	**10.61**	1.734	**11.02**	0.696	**10.61**	0.196	**11.30**	0.793	**10.42**	0.339	**11.18**	1.582	**11.01**	0.206	In Noise Range	
***Epha5^V12^***	**9.16**	0.234	**9.59**	0.570	**9.13**	0.286	**10.16**	0.592	**9.09**	0.973	**10.65**	0.793	**9.31**	0.436	**10.83**	0.321
***Epha5^V14^***	In Noise Range		In Noise Range		In Noise Range		In Noise Range		In Noise Range		In Noise Range		In Noise Range		In Noise Range	
***Epha6***	In Noise Range		In Noise Range		In Noise Range		In Noise Range		In Noise Range		In Noise Range		In Noise Range		In Noise Range	
***Epha7^V1-6^***																
***Epha8***	**10.97**	2.194	In Noise Range		**11.56**	1.252	In Noise Range		**11.62**	2.084	In Noise Range		In Noise Range		In Noise Range	
***Ephb1***	**10.64**	1.395	**11.45**	0.970	**11.49**	0.645	**11.71**	0.668	**11.10**	1.116	In Noise Range		**11.87**	0.368	In Noise Range	
***Ephb2***	**6.14**	0.300	**6.62**	0.791	**5.40**	0.196	**6.28**	0.505	**5.76**	0.190	**6.53**	0.518	**5.67**	0.427	**6.55**	0.253
***Ephb3***	**5.99**	0.167	**5.51**	0.279	**5.14**	0.179	**4.95**	0.282	**5.36**	0.304	**5.24**	0.501	**5.02**	0.308	**5.05**	0.069
***Ephb4^V1^***	**8.15**	0.249	**8.22**	0.629	**7.37**	0.307	**7.93**	0.510	**7.99**	0.203	**7.92**	0.336	**7.69**	0.325	**8.17**	0.434
***Ephb4^V2^***	**4.54**	0.178	**4.91**	0.562	**3.96**	0.270	**4.62**	0.426	**4.41**	0.144	**4.63**	0.329	**4.17**	0.053	**4.66**	0.341
***Ephb6***	**4.38**	0.219	**3.89**	0.626	**3.79**	0.138	**3.60**	0.457	**3.99**	0.201	**3.82**	0.394	**3.93**	0.004	**3.86**	0.097
***Efna1^V1^***	**4.59**	0.135	**4.59**	0.317	**4.09**	0.199	**4.28**	0.327	**4.49**	0.033	**4.18**	0.220	**4.14**	0.264	**4.35**	0.282
***Efna1^V2^***	In Noise Range		In Noise Range		In Noise Range		In Noise Range		In Noise Range		In Noise Range		In Noise Range			
***Efna2***	**10.12**	0.754	**11.00**	0.853	**9.29**	0.532	**10.60**	0.197	**10.28**	0.562	**10.99**	1.150	**9.89**	0.665	**11.54**	0.560
***Efna3^IsoA/C^***	**11.84**	0.469	In Noise Range		**10.28**	0.668	In Noise Range		**11.30**	1.049	**11.58**	0.351	**11.27**	0.583	In Noise Range	
***Efna3^IsoB/D^***	In Noise Range		In Noise Range		In Noise Range		In Noise Range		In Noise Range		In Noise Range		In Noise Range		In Noise Range	
***Efna4***	**7.07**	0.407	**7.06**	0.525	**6.25**	0.105	**6.77**	0.442	**6.90**	0.178	**6.85**	0.088	**6.46**	0.013	**6.66**	0.157
***Efna5^V1&3^***	**4.42**	0.336	**4.79**	0.780	**3.51**	0.179	**4.85**	0.529	**4.26**	0.327	**4.67**	0.333	**4.90**	0.070	**5.18**	0.170
***Efna5^V2^***	**6.24**	0.234	**6.39**	0.670	**5.52**	0.211	**6.38**	0.468	**5.98**	0.175	**6.28**	0.366	**6.13**	0.083	**6.16**	0.129
***Efnb1***	**4.42**	0.237	**4.15**	0.662	**3.87**	0.199	**4.13**	0.423	**3.95**	0.139	**3.82**	0.634	**3.83**	0.172	**4.07**	0.226
***Efnb2***	**6.49**	0.256	**6.85**	0.938	**5.92**	0.438	**6.91**	0.582	**6.04**	0.267	**6.75**	0.316	**6.04**	0.029	**6.59**	0.244
***Efnb3***	**8.38**	0.099	**9.38**	0.379	**8.03**	0.321	**8.57**	0.702	**8.56**	0.138	**9.36**	0.513	**8.55**	0.585	**9.52**	1.739

A summary of mean ΔC_q_ and standard deviation from epithelial cell readings. Expression levels are color coded in 5 categories: 3-color gradients ranging from dark to light shading indicate high to low expression, respectively; gray boxes with faded text indicate readings in the noise range (ΔC_q_ ≥ 11.9); Stippled boxes indicate undetermined or non-detected readings. Data sets with fewer than 3 replicates and/or a mean in the noise range were removed.

### 3.5 Ephrin expression changes in the aging lens epithelium

**Figure 2:**
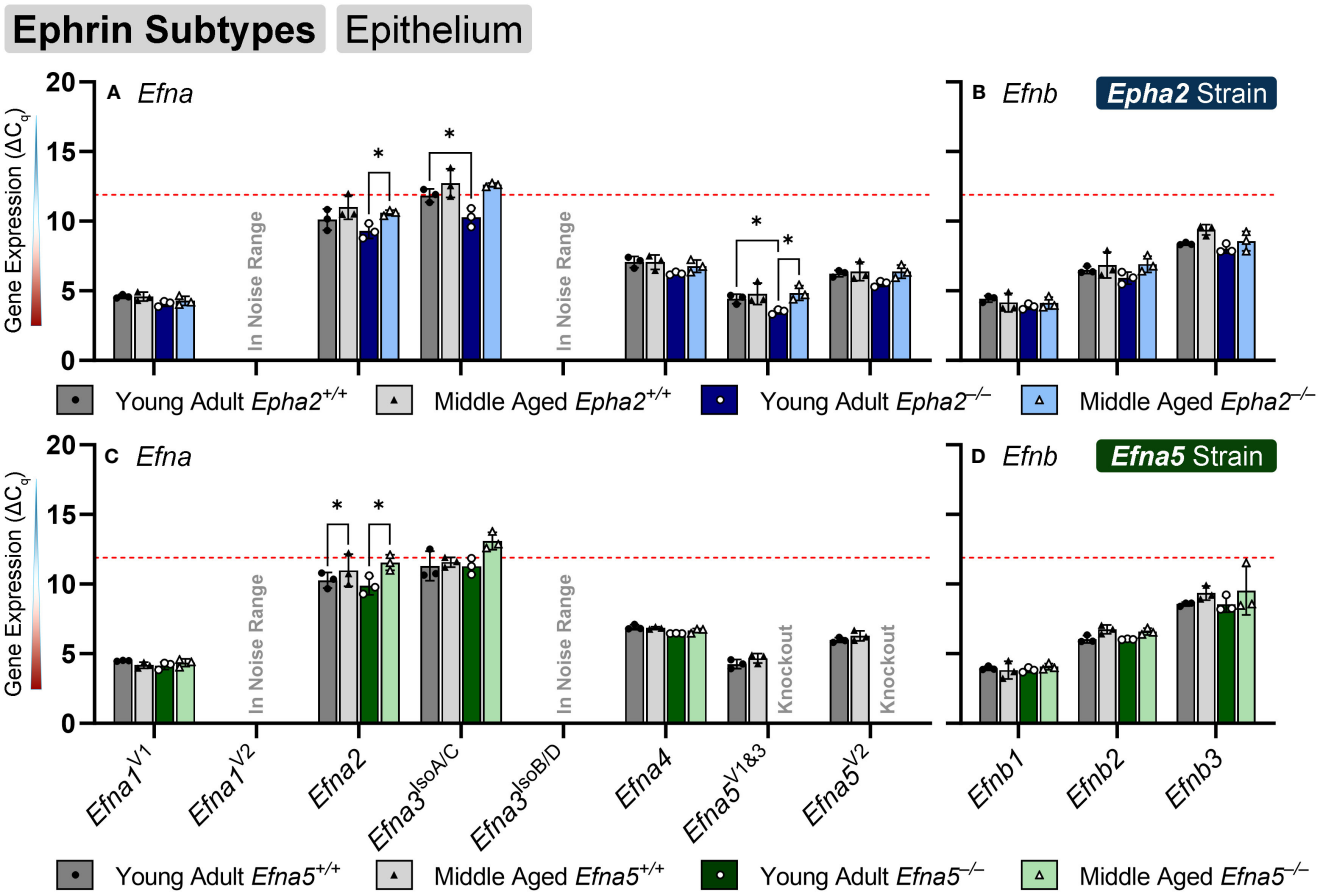
Ephrin subtypes in lens epithelium. *Efna* and *Efnb* transcripts from lens epithelial cells displayed as ΔC_q_ values. Lower ΔC_q_ values indicates higher expression. *Efna* and *Efnb* gene expression from the *Epha2* strain (panels **A**, **B**; top row; blue palette) and *Efna5* strain (panels **C**, **D**; bottom row; green palette) are displayed separately. Wild-type animals are indicated by filled symbols and grayscale bars, while knockout animals are indicated by hollow symbols and colored bars. Young-adult mice are represented by darker-shaded bars while middle-aged mice are shaded lighter. Statistically significant changes were observed in *Efna2*, and *Efna5^V1&3^* with age, and *Efna2*, *Efna3^IsoA/C^* and *Efna5^V1&3^* with genotype. Statistically significant changes with ΔC_q_ values in the noise range (ΔC_q_ ≥ 11.9), as indicated by the red dotted line, were not considered meaningful hits. Two-way ANOVA or multiple unpaired t-tests were used followed by multiple comparison correction. Benjamini-Krieger-Yekutieli-adjusted p-values (q) < 0.05 are considered discoveries and are indicated by asterisks (*).

Ephrin-A and ephrin-B ligands are encoded by *Efna* and *Efnb* genes, respectively. Within the lens epithelium, only three ephrin ligands showed significant changes in transcript levels. *Efna2*, *Efna3^IsoA/C^* (isoforms A and C), and *Efna5^V1/3^* changed significantly with expression levels in the valid range (**Figures 2A, C**, **Table 1**). *Efna2* exhibits an age-dependent decrease in both *Epha2^–/–^* and *Efna5^–/–^* epithelium (*Epha2* and *Efna5*; Y-KO vs M-KO). This age-related decrease is also seen in *Efna5^+/+^* samples (*Efna5;* Y-WT vs. M-WT) but was not significant in *Epha2^+/+^* mice (**Figure 2A**).

*Efna3* has 7 variants characterized to date that produce 4 unique protein isoforms. Variant 1 codes for isoform A, the longest of all the isoforms, which has an additional 81-amino-acid extension on the N-terminus compared to isoforms C and D. Variant 2 encodes for isoform B and also has the 81 amino acid extension; however, it lacks exon 4 (bases 591-668 in variant 1, NM_010108.1; bases 242-319 in variants 3 (NM_001377116.1), 4 (NM_001377117.1), and 5 (NM_001377118.1). Variants 3–5 encode for isoform C and only differ in the 5’ untranslated region (UTR), sharing an identical coding region. Isoform C lacks the 81 N-terminal residues but retains exon 4. Isoform D is encoded by *Efna3* variants 6 and 7, which also only differ in the 5’ UTR. Isoform D lacks the 81 amino acid N-terminal extension like isoform C and also lacks exon 4, making this the shortest isoform. An age-matched genotype difference is observed where *Efna3^IsoA/C^* expression increases between young-adult *Epha2^+/+^* and *Epha2^–/–^* mice (*Epha2*; Y-WT vs. Y-KO), but not in middle-aged mice. However, it should be noted that *Efna3^IsoA/C^* was very low expressing with a mean ΔC_q_ of 11.84, compared to the noise threshold of ΔC_q_ = 11.9.

Although ephrin-A5 is not an exclusive binding partner of EphA2, the knockout of *Epha2* resulted in an increased expression of *Efna5* variants 1 and 3 compared to wild-type in young adult mice (*Epha2*; Y-WT vs Y-KO). This elevated expression diminishes with age (*Epha2*; Y-KO vs. M-KO), becoming indistinguishable from wild-type at middle-age (*Epha2*; M-WT vs. M-KO, ns). *Efna1^V2^* and *Efna3^IsoB/D^* were both in the noise range (ΔC_q_ ≥ 11.9) and thus were not considered meaningful readings. None of the *Efnb* genes showed significant changes between test groups (**Figures 2B, D**). Although the following genes did not change with age or genotype, *Efna4*, *Efna5^V2^*, *Efnb2*, and *Efnb3* were moderately expressed, and *Efna1^V1^*, *Efna5^V1&3^*, and *Efnb1* were highly expressed (**Table 2**). Overall, 3 out of 8 ephrins showed significantly altered transcript levels in the lens epithelium (**Table 1**).

### 3.6 Eph expression changes in aging lens fibers

**Figure 3:**
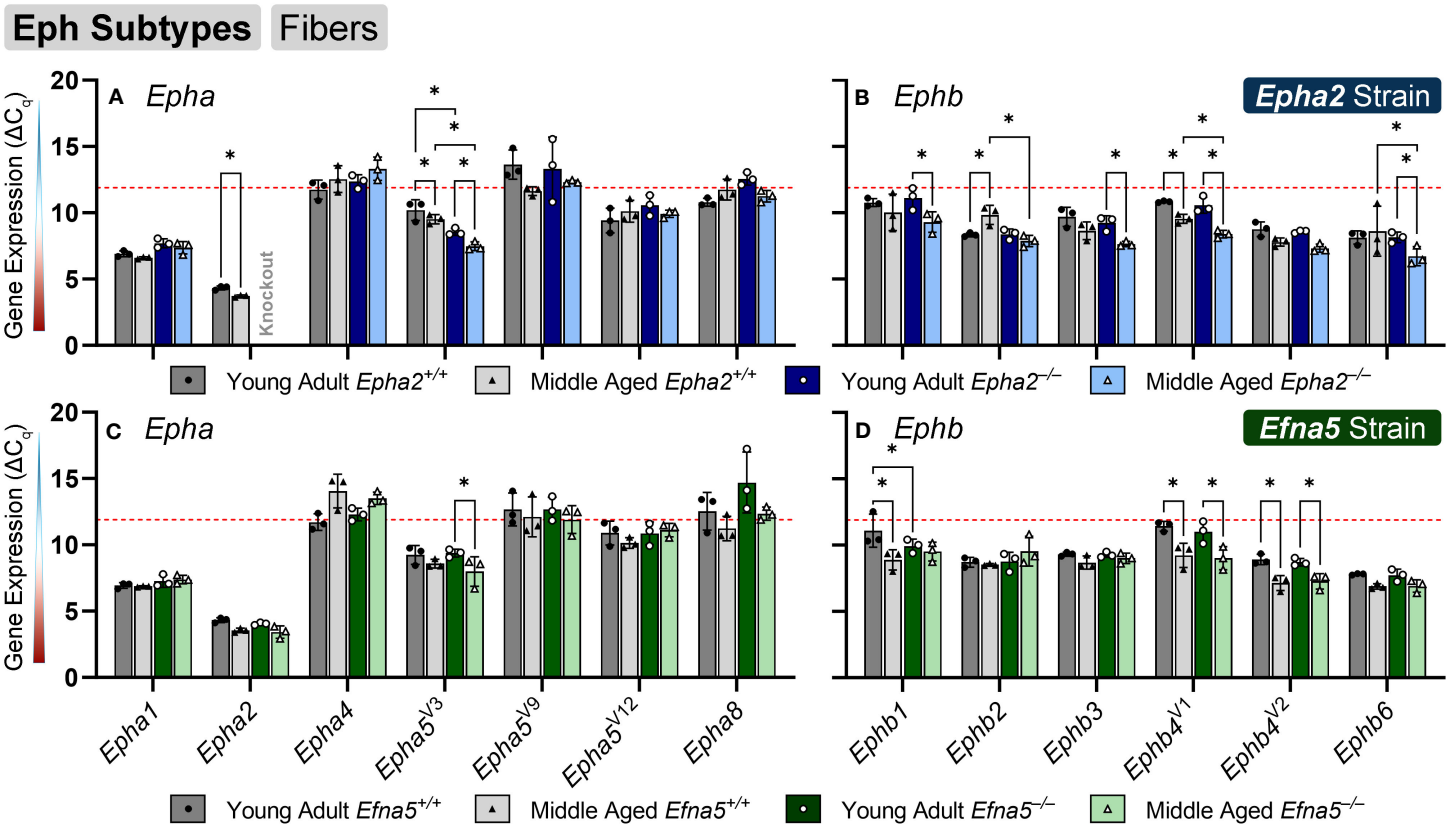
Eph subtypes in lens fibers. *Epha* and *Ephb* transcripts from lens fiber cells displayed as ΔC_q_ values. Lower ΔC_q_ values indicates higher expression. *Epha* and *Ephb* gene expression from the *Epha2* strain (panels **A**, **B**; top row; blue palette) and *Efna5* strain (panels **C**, **D**; bottom row; green palette) are displayed separately. Wild-type animals are indicated by filled symbols and grayscale bars, while knockout animals are indicated by hollow symbols and colored bars. Young-adult mice are represented by darker-shaded bars while middle-aged mice are shaded lighter. Significant changes were observed in *Epha5^V3^*, *Ephb1*, *Ephb2*, *Ephb3*, *Ephb4*, *Ephb5*, and *Ephb6* with age and genotype. Statistically significant changes with ΔC_q_ values in the noise range (ΔC_q_ ≥ 11.9), as indicated by the red dotted line, were not considered meaningful hits. Two-way ANOVA or multiple unpaired t-tests were used followed by multiple comparison correction. Benjamini-Krieger-Yekutieli-adjusted p-values (q) < 0.05 are considered discoveries and are indicated by asterisks (*).

In isolated lens fibers, *Epha2*, *Epha5^V3^*, and all the *Ephb* subtypes (*Ephb1-4*, *Ephb6*) showed altered gene expression either with age or genotype (**Figure 3**, **Table 3**). *Epha2* levels increased with age in *Epha2* ^+/+^ fibers (*Epha2*; Y-WT vs. M-WT) but showed no changes in *Efna5* mice (**Figure 3C**). *Epha5^V3^* showed both age- and genotype-dependent increases in expression. *Epha5^V3^* transcript levels increased in aging mice regardless of genotype (*Epha2*; Y-WT vs. M-WT; Y-KO vs. M-KO). Moreover, genotype differences were observed where *Epha5^V3^* transcript levels were increased in *Epha2^–/–^* lens fibers compared to wild-type mice at both age groups (*Epha2*; Y-WT vs. Y-KO; M-WT vs. M-KO). In contrast, a strain difference is observed where *Epha5^V3^* only increased in *Efna5^–/–^* fibers with aging (*Efna5*; Y-KO vs. M-KO) but not in aging *Efna5^+/+^* mice (*Efna5*; Y-WT vs. M-WT, ns) or in response to *Efna5* knockout (*Efna5*; Y-WT vs. Y-KO, ns; M-WT vs. M-KO, ns).

All the tested *Ephb* genes exhibited a significant change in the lens fibers (**Figures 3B, D**); however strain-dependent changes are evident here. *Ephb1* expression did not change in *Epha2* ^+/+^ WT lens fibers (*Epha2;* Y-WT vs. M-WT, ns), but showed a marked increase with age in *Epha2^–/–^* fibers (*Epha2;* Y-KO vs. M-KO). *Ephb1* undergoes a strain-dependent increase in aging wild-type lenses (*Efna5;* Y-WT vs. M-WT) of *Efna5^+/+^* mice, unlike the *Epha2* strain. A further genotype difference is seen where *Ephb1* increases with the knockout of *Efna5* in young-adult mice (*Efna5;* Y-WT vs. Y-KO). In the *Epha2* strain, *Ephb2* transcript levels decrease between young-adult and middle-aged wild-type mice (*Epha2*; Y-WT vs. M-WT) but remains steady in middle-aged *Epha2^–/–^* mice (*Epha2*; Y-KO vs. M-KO). The level of *Ephb2* expression is significantly higher in middle-aged *Epha2^–/–^* fibers compared to middle-aged wild-type samples (*Epha2*; M-WT vs. M-KO). *Ephb2* is the only instance of decreased expression with age observed within lens fibers in this screen. *Ephb3* only showed an age-related increase in *Epha2^–/–^* fibers (*Epha2*; Y-KO vs. M-KO).

*Ephb4^V1^* increased with age in both strains (*Epha2* and *Efna5*; Y-WT vs. M-WT; Y-KO vs. M-KO), but also showed an increased expression in middle-aged *Epha2^–/–^* samples (*Epha2*; M-WT vs. M-KO). In contrast, *Ephb4^V2^* increased with age only in the *Efna5* strain (*Efna5*; Y-WT vs. M-WT; Y-KO vs. M-KO). *Ephb6* showed a significant increase in the middle-aged *Epha2^–/–^* fibers compared to its wild-type counterpart (*Epha2*; M-WT vs. M-KO) and its young-adult knockout control (*Epha2*; Y-KO vs. M-KO).

Of all the Eph changes in aging lens fibers, *Ephb1* was the only target that changed concomitantly with the knockout of *Efna5* (**Figure 3D**). *Epha5^V3^*, *Ephb2*, Ephb4^V1^, and *Ephb6* all showed increases in expression in *Epha2^–/–^* samples compared to their age-matched *Epha2^+/+^* control (**Table 3**). *Epha3*, *Epha4*, *Epha^V9^*, *Epha5^V14^*, *Epha6*, and all *Epha7* variants (1–6) were in the noise range or did not produce readable signal due to low transcript levels. *Epha8* was above the threshold in the *Efna5* strain and was low expressing in the *Epha2* strain. *Epha5^V12^*, *Epha8*, *Ephb1*, and *Ephb4^V1^* were all low expressing, and *Epha1*, *Epha5^V3^*, *Ephb2*, *Ephb3*, *Ephb4^V2^*, and *Ephb6* were all moderately expressing (**Table 4**). Out of all the Eph receptor genes in the fibers, *Epha2* was the only highly expressed target with a mean ΔC_q_ of 3.9. Overall, changes in *Eph* gene expression are more numerous in the lens fibers compared to the epithelium, with 7 of 14 characterized Ephs exhibiting a significant change.

**Table 3:** Lens fiber dynamics summary.

Expression Change Summary	*Epha2* Lens Fiber				*Efna5* Lens Fiber			
	Y WT vs M WT	Y KO vs M KO	Y WT vs Y KO	M WT vs M KO	Y WT vs M WT	Y KO vs M KO	Y WT vs Y KO	M WT vs M KO
***Epha1***	** **	** **	** **	** **	** **	** **	** **	** **
***Epha2***	▲	** **	** **	** **	** **	** **	** **	** **
***Epha5^V3^***	▲	▲	▲	▲	** **	▲	** **	** **
***Epha5^V12^***	** **	** **	** **	** **	** **	** **	** **	** **
***Ephb1***	** **	▲	** **	** **	▲	** **	▲	** **
***Ephb2***	▼	** **	** **	▲	** **	** **	** **	** **
***Ephb3***	** **	▲	** **	** **	** **	** **	** **	** **
***Ephb4^V1^***	▲	▲	** **	▲	▲	▲	** **	** **
***Ephb4^V2^***	** **	** **	** **	** **	▲	▲	** **	** **
***Ephb6***	** **	▲	** **	▲	** **	** **	** **	** **
***Efna1***	▲	▲	** **	** **	▲	▲	** **	** **
***Efna2***	** **	** **	** **	** **	** **	** **	** **	** **
***Efna3^IsoA/C^***	** **	** **	** **	** **	** **	** **	** **	** **
***Efna5^V1&3^***	** **	** **	** **	** **	** **	** **	** **	** **
***Efna5^V2^***	** **	** **	** **	** **	▲	** **	** **	** **
***Efnb1***	▲	▲	▲	▲	▲	▲	** **	** **
***Efnb2***	▲	▲	** **		▲	▲	** **	** **

A summary table of significant gene expression hits in fiber cells across *Epha2* and *Efna5* strains. Young-adult (Y) vs. middle-aged (M) comparisons (columns 1 and 2 in each set) indicate age-related changes, while wild-type (WT) vs knockout (KO) comparisons in columns 3 and 4 indicate a genotype-related change. Gene upregulation (▲) is indicated in black, while downregulation (▼) is shown in red. Genes that did not change across any of the characterized groups are not included in the table.

### 3.7 Ephrin expression changes in aging lens fibers

**Figure 4:**
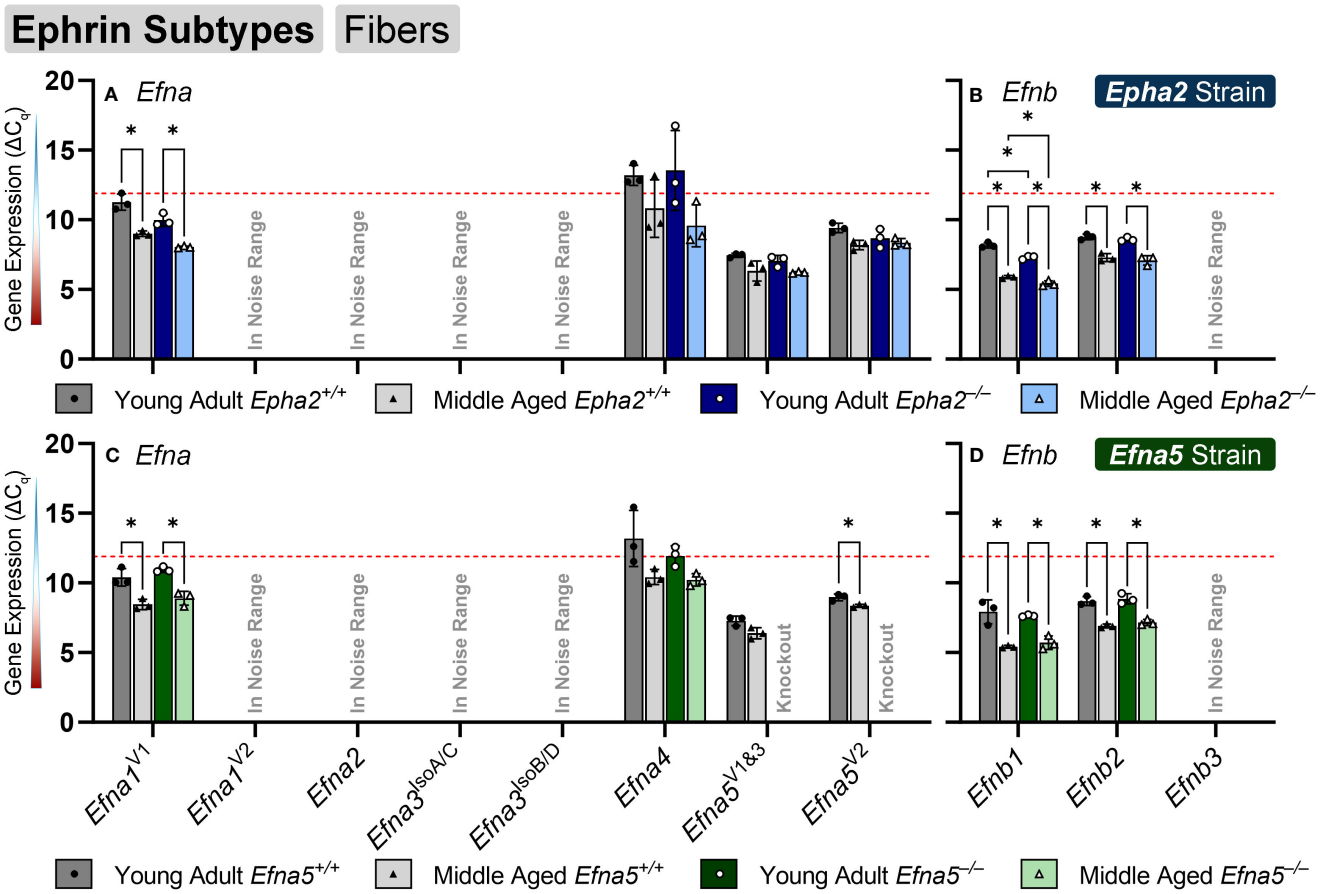
Ephrin subtypes in lens fibers. *Efna* and *Efnb* transcripts from lens fiber cells displayed as ΔC_q_ values. Lower ΔC_q_ values indicates higher expression. *Efna* and *Efnb* gene expression from the *Epha2* strain (panels **A**, **B**; top row; blue palette) and *Efna5* strain (panels **C**, **D**; bottom row; green palette) are displayed separately. Wild-type animals are indicated by filled symbols and grayscale bars, while knockout animals are indicated by hollow symbols and colored bars. Young-adult mice are represented by darker-shaded bars while middle-aged mice are shaded lighter. Significant changes were observed in *Efna1^V1^*, *Efna4*, *Efnb1*, and *Efnb2* with age and genotype. *Efna1^V1^*, *Efnb1*, and *Efnb2* all increased with age, however *Efna1^V1^* also showed an increased expression in *Epha2* ^–/–^ fiber cells compared to the age-matched *Epha2* ^+/+^ samples. A genotype difference was also observed with increased Efna4 expression in middle-aged *Epha2* ^–/–^ and *Epha2* ^+/+^ mice. Statistically significant changes with ΔC_q_ values in the noise range (ΔC_q_ ≥ 11.9), as indicated by the red dotted line, were not considered meaningful hits. Two-way ANOVA or multiple unpaired t-tests were used followed by multiple comparison correction. Benjamini-Krieger-Yekutieli-adjusted p-values (q) < 0.05 are considered discoveries and are indicated by asterisks (*).

In the isolated lens fibers, *Efna1^V1^*, *Efna5^V2^*, *Efnb1*, and *Efnb2* showed significant changes with age or genotype (**Figure 4**, **Table 4**). *Efna1^V1^* increased with age across both strains in wild-type and knockout lens fibers (*Epha2* and *Efna5*; Y-WT vs. M-WT; Y-KO vs. M-KO). A genotype difference is observed as young adult *Epha2^–/–^* fibers showed significantly higher expression of *Efna1^V1^* than the wild-type controls (*Epha2*; Y-KO vs. M-KO). *Efna5^V2^* increased with age in wild-type *Efna5* mice (*Efna5*; Y-WT vs. M-WT), but not in the *Epha2* strain (**Figures 4A, C**). In contrast, *Efna5^V1/3^* did not change in the fibers. Targets in the noise range included *Efna1^V2^*, *Efna2*, *Efna3^IsoA/C^*, *Efna3^IsoB/D^*, and *Efnb3*. These targets either returned low transcript levels indistinguishable from noise or failed to produce readable signals. *Efnb1* and *Efnb2* increased with age regardless of genotype across both strains (*Epha2* and *Efna5*; Y-WT vs. M-WT; Y-KO vs. M-KO). However, *Efnb1* also increased in *Epha2* knockouts compared to their wild-type counterparts (*Epha2*; Y-WT vs. Y-KO; M-WT vs. M-KO). In the lens fibers, *Efna1* and *Efna4* were lowly expressed while *Efna5^V1&3^*, *Efna5^V2^*, *Efnb1*, and *Efnb2* were moderately expressed. There were no ephrins considered highly expressed in the lens fibers (**Table 3**).

**Table 4:** Lens fiber expression table.

Mean ΔC_q_ Summary	*Epha2* Lens Fiber								*Efna5* Lens Fiber							
	Y WT		M WT		Y KO		M KO		Y WT		M WT		Y KO		M KO	
	Mean	St.Dev.	Mean	St.Dev.	Mean	St.Dev.	Mean	St.Dev.	Mean	St.Dev.	Mean	St.Dev.	Mean	St.Dev.	Mean	St.Dev.
***Epha1***	**6.91**	0.223	**6.61**	0.088	**7.71**	0.334	**7.36**	0.473	**6.93**	0.211	**6.87**	0.042	**7.26**	0.463	**7.39**	0.313
***Epha2***	**4.34**	0.146	**3.72**	0.089					**4.32**	0.177	**3.55**	0.162	**4.05**	0.096	**3.42**	0.459
***Epha3***	In Noise Range		** **		In Noise Range											
***Epha4***	**11.75**	0.720	In Noise Range		In Noise Range		In Noise Range		**11.72**	0.618	In Noise Range		In Noise Range		In Noise Range	
***Epha5^V3^***	**10.20**	0.776	**9.52**	0.350	**8.56**	0.270	**7.48**	0.327	**9.24**	0.708	**8.58**	0.328	**9.38**	0.298	**7.99**	1.122
***Epha5^V9^***	In Noise Range		**11.63**	0.327	In Noise Range		In Noise Range		In Noise Range		In Noise Range		In Noise Range		**11.90**	1.050
***Epha5^V12^***	**9.41**	0.930	**10.12**	0.832	**10.57**	0.775	**9.92**	0.272	**10.90**	0.902	**10.14**	0.406	**10.86**	0.858	**11.12**	0.507
***Epha5^V14^***	In Noise Range		In Noise Range		In Noise Range		In Noise Range				In Noise Range		In Noise Range		** **	
***Epha6***	** **		** **				** **				** **					
***Epha7^V1-6^***	** **		** **								** **					
***Epha8***	**10.79**	0.298	**11.74**	0.788	In Noise Range		**11.25**	0.440	In Noise Range		**11.23**	0.925	In Noise Range		In Noise Range	
***Ephb1***	**10.77**	0.305	**10.03**	1.375	**11.11**	0.837	**9.30**	0.779	**11.09**	1.245	**8.90**	0.763	**9.93**	0.512	**9.52**	0.712
***Ephb2***	**8.34**	0.144	**9.84**	0.720	**8.38**	0.391	**7.86**	0.424	**8.71**	0.369	**8.52**	0.088	**8.76**	0.702	**9.55**	1.124
***Ephb3***	**9.70**	0.725	**8.64**	0.662	**9.23**	0.551	**7.64**	0.196	**9.31**	0.149	**8.69**	0.494	**9.27**	0.201	**9.01**	0.393
***Ephb4^V1^***	**10.83**	0.062	**9.55**	0.343	**10.57**	0.613	**8.39**	0.292	**11.42**	0.363	**9.22**	0.912	**11.00**	0.846	**9.01**	0.866
***Ephb4^V2^***	**8.77**	0.553	**7.79**	0.313	**8.58**	0.098	**7.33**	0.317	**8.91**	0.391	**7.14**	0.566	**8.71**	0.289	**7.27**	0.576
***Ephb6***	**8.13**	0.500	**8.61**	1.884	**8.14**	0.404	**6.73**	0.713	**7.81**	0.055	**6.89**	0.207	**7.73**	0.465	**6.92**	0.475
***Efna1^V1^***	**11.27**	0.580	**9.01**	0.199	**9.98**	0.450	**8.04**	0.070	**10.40**	0.631	**8.46**	0.376	**10.95**	0.196	**8.89**	0.496
***Efna1^V2^***	In Noise Range		** **				** **				** **					
***Efna2***	In Noise Range		** **		In Noise Range		In Noise Range		In Noise Range				In Noise Range		** **	
***Efna3^IsoA/C^***	** **		** **		In Noise Range		** **				In Noise Range				In Noise Range	
***Efna3^IsoB/D^***	** **				In Noise Range		** **				** **					
***Efna4***	In Noise Range		**10.81**	2.062	In Noise Range		**9.58**	1.525	In Noise Range		**10.42**	0.540	In Noise Range		**10.22**	0.450
***Efna5^V1&3^***	**7.45**	0.154	**6.32**	0.720	**7.05**	0.395	**6.22**	0.069	**7.29**	0.339	**6.39**	0.395	**7.73**	0.470	**6.79**	0.291
***Efna5^V2^***	**9.43**	0.338	**8.19**	0.334	**8.68**	0.669	**8.36**	0.296	**8.97**	0.241	**8.36**	0.106	**8.82**	0.206	**8.14**	0.195
***Efnb1***	**8.17**	0.193	**5.89**	0.104	**7.30**	0.126	**5.43**	0.206	**7.92**	0.853	**5.42**	0.089	**7.63**	0.080	**5.71**	0.483
***Efnb2***	**8.77**	0.202	**7.29**	0.294	**8.59**	0.155	**7.09**	0.340	**8.69**	0.317	**6.91**	0.144	**8.85**	0.365	**7.16**	0.219
***Efnb3***	In Noise Range		In Noise Range		In Noise Range		In Noise Range		In Noise Range		** **		** **			

A summary of mean ΔC_q_ and standard deviation from fiber cell readings. Expression levels are color coded in 5 categories: 3-color gradients ranging from dark to light shading indicate high to low expression, respectively; gray boxes with faded text indicate readings in the noise range (ΔC_q_ ≥ 11.9); Stippled boxes indicate undetermined or non-detected readings. Data sets with fewer than 3 replicates and/or a mean in the noise range were removed

In summary, within the lens epithelium, *Epha1*, *Epha5^V3^*, *Epha5^V12^*, *Efna2*, *Efna3^IsoA/C^*, and *Efna5^V1&3^*, showed significant changes with age. *Efna3^IsoA/C^, and Efna5* were the only targets that changed with genotype, increasing in middle-aged *Efna5^–/–^* and young adult *Epha2^–/–^* epithelium, respectively, compared to their age-matched wild-type samples. In the lens fibers, *Epha2*, *Epha5^V3^*, all the *Ephb* genes (*Ephb1*-*Ephb6*), *Efna1^V1^*, *Efna5^V2^*, *Efnb1*, and *Efnb2* demonstrated age-related changes, some of which were strain-dependent. *Epha5^V3^*, *Ephb2*, *Ephb4^V1^*, *Ephb6*, and *Efna4* exhibited expression changes concomitant with genotype, notably all in *Epha2^–/–^* samples. *Ephb1* was the only target that increased in *Efna5* knockout fibers compared to the wild-type control (**Figure 4D**). Moreover, *Ephb2* was the only target that decreased with age in the lens fibers.

## 4 Discussion

Our data show a constellation of gene expression changes between young-adult and middle-aged lenses, as well as changes concomitant with the disruption of *Epha2* or *Efna5* in both cell types of the ocular lens. While transcript-level changes were observed in both the epithelium and isolated fibers, the majority of altered gene expression was observed in the lens fiber cells. Of the genes and variants screened, 6 out of 33 genes and variants screened in the lens epithelium were considered hits, as opposed to 12 out of 33 in the fiber cells. Collapsing these metrics to gene families, 3 of 14 Ephs and 3 of 8 ephrins were considered hits in the epithelium, while 7 of 14 Ephs and 4 of 8 ephrins were regarded as hits in the lens fibers. Our previous work found that 12 of 14 Ephs and 8 of 8 ephrins were present in the ocular mouse lens (27). This work has characterized that, of these previous hits, *Epha3*, *Epha5^V14^*, *Epha7^V1–6^*, *Efna1^V2^*, and *Efna3^IsoB/D^* are very low expressing (in the noise range or not detected) and confirmed the non-detection of *Epha6* in both the epithelium and isolated fibers (**Tables 2**, **4**). Moreover, these data are generally consistent with the previously reported Affymetrix 430 2.0 data from P28 epithelium and P56 whole lenses obtained from the iSyTE 2.0 database (27). Here, we can prioritize targets based on changes with age, genotype, or expression level, allowing the finer focusing of future studies.

In order to properly control for variation from tissues and genetic knockouts, we screened for a reference gene that is consistent across all of our determined test conditions. While ß-actin is commonly used as an endogenous control, *Epha2* knockout mice exhibited dysregulation of actin and actin-binding proteins (20). Therefore, a non-cytoskeletal control had to be used in this study. This is an important step if the reference gene has not already been characterized in each disease or transgenic model.

In determining meaningful hits from this set of experiments, several different criteria were considered. Data sets with a mean ΔC_q_ ≥ 11.9 were regarded as noise based on the threshold obtained from the *Epha2* in *Epha2^–/–^* samples. Although low-expressing targets can play important biological roles, these were considered low priority due to additional steps required to differentiate this signal from noise. Moreover, samples with wild-type controls that did not match across the *Epha2* and *Efna5* strains were considered lower priority due to potential strain differences. In determining statistical significance, a false discovery rate (FDR) approach was chosen over a family-wise error rate (FWER), allowing for more leniency towards type I errors (false positives) (39–41). This was considered more useful than a stricter FWER approach in this context as the goal of these experiments was to establish meaningful leads rather than a mechanistic or diagnostic investigation.

In the traditional PCR screening of Ephs and ephrins in the lens, *Epha7^V1^*, was identified to be present in wild-type mice, but not in *Epha2^–/–^* or *Efna5^–/–^* samples, identifying this target as a potential lead (27). In the current study, *Epha7^V1^* was not detected in any of the samples, indicating the expression levels may be too low to easily pursue. Previously, *Efna1^V2^* was shown to be present in lens epithelium but not isolated fibers. Here, *Efna1^V2^* resulted in 12/24 non-detects in epithelial samples, and the remaining 12/24 were in the noise range. In the lens fibers, only 1/24 samples yielded a detectable signal, and that reading was in the noise range, consistent with the qualitative PCR data (27). *Efnb3* was also previously detected in the epithelium but not fibers. Here, *Efnb3* was consistently detected in the lens epithelium with 24/24 valid readings, but not in the isolated lens fibers where 6/24 readings were detected, of which 4 were in the noise range.

Previous work successfully narrowed down the 14 variants of *Epha5* to variants 3, 9, 12, and 14 that were present in the lens. This current work further narrowed down these variants, finding that *Epha5^V14^* produced a signal indistinguishable from noise. The remaining variants 3, 9, and 12 were present in both lens epithelium and fibers. *Epha5^V9^* was low expressing and did not show appreciable change across any of the test conditions in this screen. However, *Epha5^V3^* expression showed opposite trends between tissue compartments, decreasing with age in the epithelium (*Efna5*; Y-KO vs. M-KO) and increasing with age in the isolated lens fibers (*Efna5*; Y-KO vs. M-KO). The opposing trends between tissues and the increase of expression in the *Epha2* fibers may indicate a compensatory or related physiological role linked to the knockout of *Epha2* or *Efna5*. Meanwhile, *Epha5^V12^* decreased with age only in the epithelium of both wild-type and *Efna5^–/–^* mice, but not in the *Epha2* strain. *Epha5* was previously observed to be highly expressed in the lenses of embryonic mice, second only to *Epha2* based on iSyTE analysis, and the EphA5 receptor is known to bind to ephrin-A5. Moreover, *Epha5* expression was decreased in *Mafg^–/–^*;*Mafk^–/–^* double knockout mice, a model that exhibits cataracts after 4 months of age (42). These findings highlight potential roles of *Epha5^V3^* and *Epha5^V12^* that differ between the epithelium and isolated fibers, and also between closely related variants. Further study into the roles of *Epha5* variants in the epithelium vs. bulk fiber mass may be a promising avenue into revealing the mechanisms of cataractogenesis.

Finding the binding partners of EphA2 and ephrin-A5, even with a putatively narrowed list of hits, can be a time- and resource-intensive process. Given that EphAs typically bind to ephrin-As, and EphBs typically bind to ephrin-Bs, targets can be tentatively filtered in this way; however, there are several exceptions to this paradigm that must be considered. Although the roles of other Ephs and ephrins in the lens remain unclear, interactions between EphA2, ephrin-A5, and other binding partners have been reported in other tissues. Several studies have been conducted investigating the interactions between EphA2 and ephrin-A1, showing roles in processes ranging from angiogenesis, proliferation, cell migration, and adhesion via an assortment of secondary messengers (43–52). In this study, *Efna1* is a high-expressing ligand in the lens epithelium and a moderately-expressing target in the isolated fibers. When *Epha2* is knocked out, the expression level of *Efna1* increases significantly in both the epithelium and fibers, suggesting a possible compensatory mechanism. The previously observed interactions between EphA2 and ephrin-A1 make this a promising interaction to further investigate at the protein level.

Beyond binding with several ephrin ligands, EphA2 has also been shown to interact with other Eph receptors as well. In the PC3 human prostate cancer cell line, EphA2 coimmunoprecipitated with EphB2 via the ligand binding domain, showing a co-clustering of these receptors that may differentially affect the Eph-ephrin signaling landscape based on the present heteromers (53). Here, a marked increase in *Ephb2* expression is observed in middle-aged *Epha2* ^–/–^ fibers compared to its middle-aged wild-type counterpart, and this change is not seen in *Efna5^–/–^* samples.

Like EphA2, ephrin-A5 has also been shown to interact with several other Eph receptors. Ephrin-A5 interaction with *EphA5* has been shown to promote synaptogenesis through activation of voltage-gated calcium channels, promoting downstream signaling through protein kinase A (54). While calcium is a broad secondary messenger, calcium-dependent or calcium-modulated targets play important roles in the lens and cataract formation, including cadherins, calcium-activated proteases (calpain), aquaporin-0, and connexins (4, 55–58). These calcium-dependent targets have been observed to be dysregulated in cataractous lenses, suggesting that disruption of ephrin-A5 and EphA5 interactions could play a role in these mechanisms of cataractogenesis. Moreover, ephrin-A5 and *EphB2* have been shown to interact with high affinity, and here, *Ephb2* is observed at moderate to high expression levels in the lens epithelium and moderate levels in the fibers (10). Between activating protein kinase A and increasing calcium influxes, the downstream effectors of ephrin-A5 are vast. Considering interactions with EphA5 and EphB2, identifying other Ephs and ephrins as interacting partners of ephrin-A5 may provide leads for a more targeted search for calcium- and phosphorylation-dependent mechanisms of cataractogenesis.

As the roles of Ephs and ephrins are characterized in other tissues, these data can be used to help direct the focus of future studies in the lens. Several transgenic models are available for Ephs and ephrins, however, little phenotypic data of the lens has been reported to date in these models. Reporting these phenotypes can also help identify Ephs and ephrins of interest and rapidly expand the compendium of relevant targets. In the context of previous studies investigating EphA2 and ephrin-A5, the spatial and temporal expression data presented here may help inform the choice of targets of interest of lens homeostasis and cataractogenesis in the lens,

Although mRNA transcripts are well understood in the central dogma of molecular biology to encode the translation of protein, the correlation between transcript and protein levels is weak (59–62). This is observed here, where the data presented in this study indicates an increase in *Epha2* transcripts, however, western blot data shows a decrease in EphA2 protein between 2 weeks and 5 months of age in mice (6). In order to pursue meaningful hits, quantification of the relevant proteins should be performed to ensure that the target is present in a given strain before undergoing an in-depth physiological assessment. Moreover, in our endeavor to identify binding partners of EphA2 and ephrin-A5, characterizing the localization of the target of interest will be a necessary step. This is due to the observation that EphA2 sequesters mostly in the fiber cells and while ephrin-A5 is mostly present in the epithelial cells, resulting in a spatial barrier of interaction (5). Additionally, the loss of nuclei and other organelles in the nuclear fiber cells presumably limits transcriptomics data to the peripheral fiber cells. Therefore, characterization of these targets at the protein level will be an important step in informing their role in the lens, particularly in the lens nucleus.

Currently, the primary interacting partners of EphA2 and ephrin-A5 in the lens are not known. Here, we present the characterization of a dynamic gene network, changing concomitantly within two transgenic models. These data provide an informed prioritization of targets to pursue in upcoming mechanistic studies to determine how EphA2 and ephrin-A5 maintain lens homeostasis and lens transparency. This perspective on gene network changes in these transgenic cataract models has helped focus the Eph-ephrin search to fewer, manageable targets to pursue in future studies.

## Data availability statement

The original contributions presented in the study are included in the **Supplementary Material**, further inquiries can be directed to the corresponding author.

## Ethics statement

The animal study was approved by The Bloomington Institutional Animal Care and Use Committee. The study was conducted in accordance with the local legislation and institutional requirements.

## Author contributions

PH: Conceptualization, Data curation, Formal analysis, Investigation, Methodology, Project administration, Software, Validation, Visualization, Writing – original draft, Writing – review & editing. CC: Conceptualization, Funding acquisition, Investigation, Methodology, Project administration, Resources, Supervision, Visualization, Writing – review & editing.

## Supplementary material

Supplementary Figure 1
*Epha7* probe design strategy. Probe permutations used to target *Epha7* variants 1–6. Each bar represents the sequence ranging from the end of exon 7 to beginning of exon 10. The primer permutations and expected amplicon sizes are listed in the bottom panel.

Supplementary Figure 2 Endogenous gene panel. Quantification cycle (C_q_) values of endogenous reference genes from the ocular lenses of young-adult (6-week-old) mice. The chosen reference gene, peptidylprolyl isomerase A (*Ppia*) is indicated by a red box. Each of the 8 test groups denoted were tested in triplicate (n=3 per group).

Supplementary Figure 3 Peptidylprolyl isomerase A distribution. Quantification cycle (C_q_) values of the endogenous internal control gene, peptidylprolyl isomerase a (*Ppia*) across biological groups. These are aggregates of all the readings taken from 48 biological samples across all the reported assays (n=1584).

Supplementary Table 1 Primer and probe library. The primers and probes used in this study with sequences of custom probes and assay IDs of commercial probes are provided. For custom probes, the exon-spanning oligonucleotide is indicated as the forward primer (F), reverse primer (R), or TaqMan probe (P). The sequencing primers used for Efna5–/– validation are included as the last entry in the table. Alternative exons are designated as 'a-d' using variant 1 as reference (except for Epha5, for which variant 3 was used as reference). Greyed out exons indicate untranslated regions (UTRs).

Supplementary Table 2 Lens epithelium statistics. A compiled table of two-way ANVOAs and multiple t-tests with two-stage linear step-up procedure of Benjamini, Krieger, and Yekutieli multiple comparison correction. Significant hits are indicated as discoveries.

Supplementary Table 3 Lens fiber statistics. A compiled table of two-way ANVOAs and multiple t-tests with two-stage linear step-up procedure of Benjamini, Krieger, and Yekutieli multiple comparison correction. Significant hits are indicated as discoveries.

Supplementary Table 4 Assay data. Sorted assay data containing Cq and ΔCq values for each gene of interest.
