# Supplementary figures and images for: Spatial-temporal comparison of Eph/Ephrin gene expression in ocular lenses from aging and knockout mice
Source: Front Ophthalmol (Lausanne). 2024 Jun 4;4:1410860. doi: 10.3389/fopht.2024.1410860 (PMC11182306; doi:10.3389/fopht.2024.1410860)

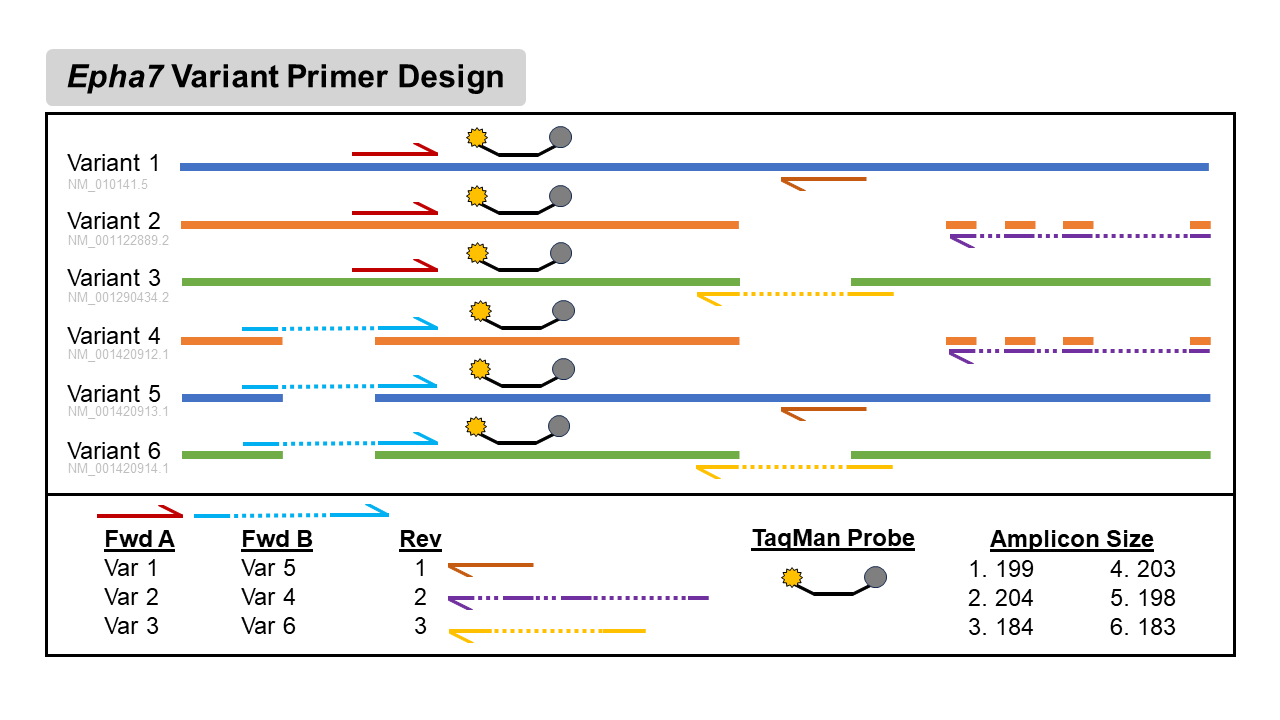

Supplement: Supplementary Figure 1 — Epha7 probe design strategy. Probe permutations used to target Epha7 variants 1–6. Each bar represents the sequence ranging from the end of exon 7 to beginning of exon 10. The primer permutations and expected amplicon sizes are listed in the bottom panel. [file Image_1.tif]

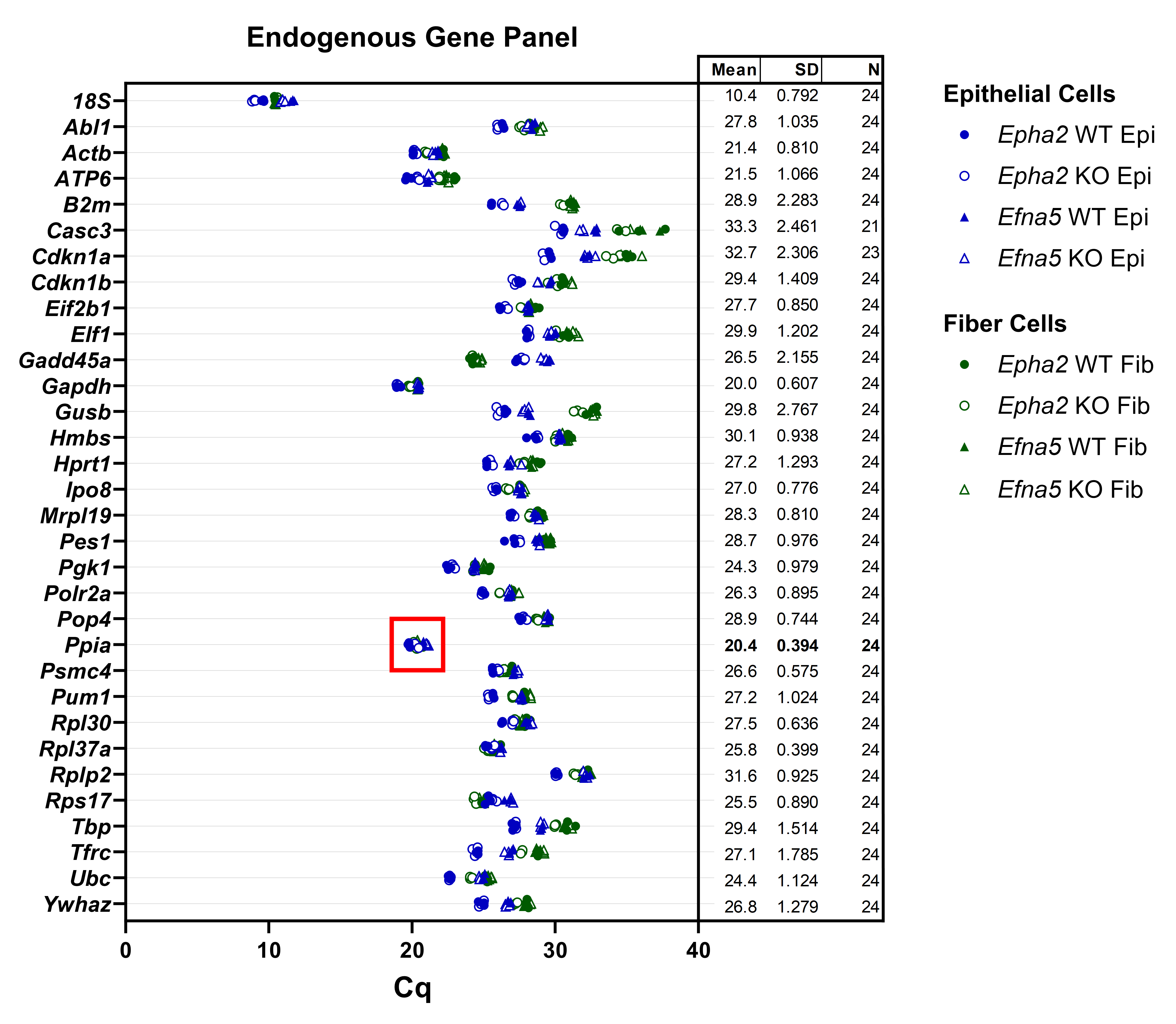

Supplement: Supplementary Figure 2 — Endogenous gene panel. Quantification cycle (Cq) values of endogenous reference genes from the ocular lenses of young-adult (6-week-old) mice. The chosen reference gene, peptidylprolyl isomerase A (Ppia) is indicated by a red box. Each of the 8 test groups denoted were tested in triplicate (n=3 per group). [file Image_2.tif]

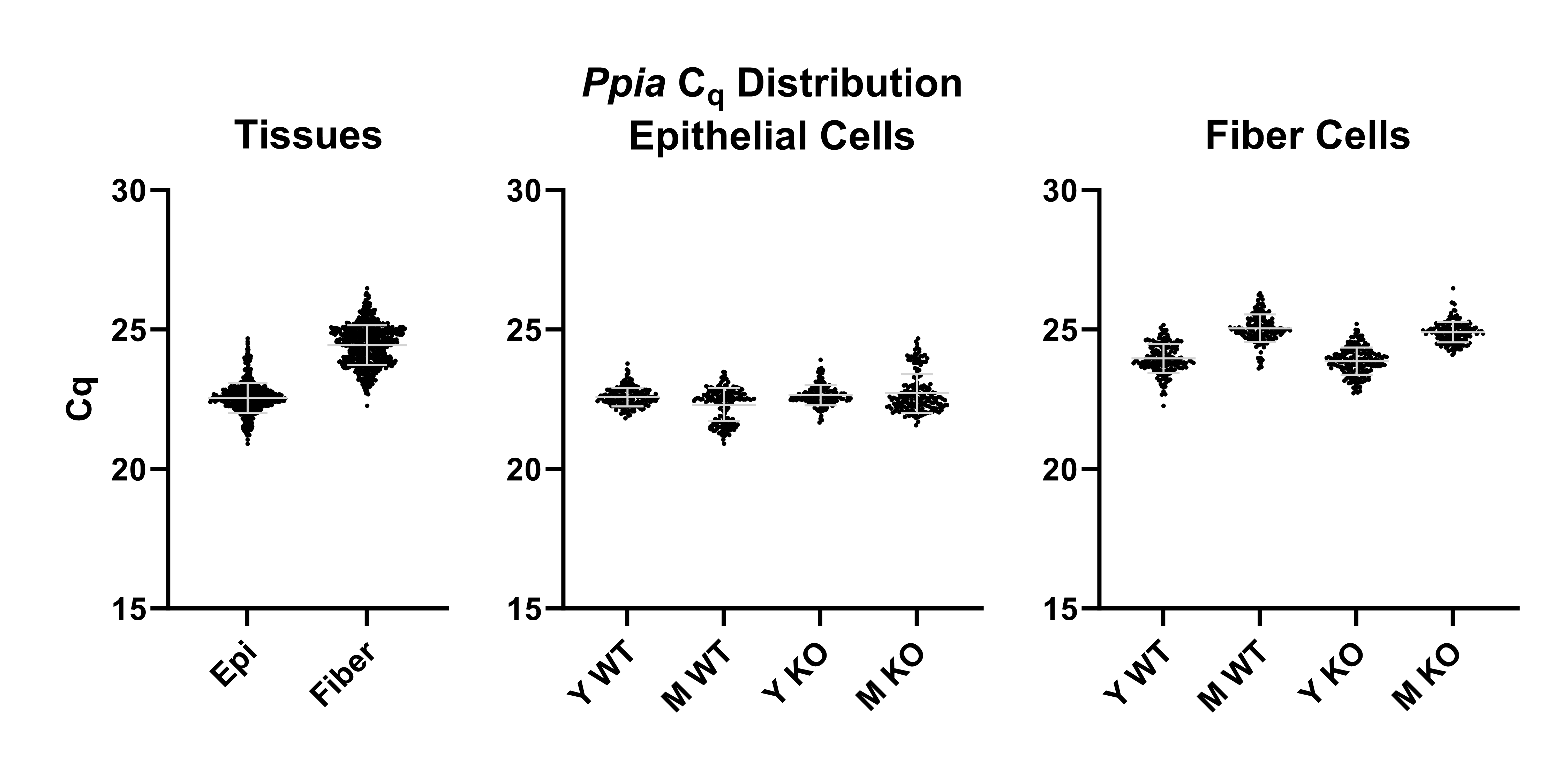

Supplement: Supplementary Figure 3 — Peptidylprolyl isomerase A distribution. Quantification cycle (Cq) values of the endogenous internal control gene, peptidylprolyl isomerase a (Ppia) across biological groups. These are aggregates of all the readings taken from 48 biological samples across all the reported assays (n=1584). [file Image_3.tif]
